# Water Environmental Capacity Analysis of Taihu Lake and Parameter Estimation Based on the Integration of the Inverse Method and Bayesian Modeling

**DOI:** 10.3390/ijerph121012212

**Published:** 2015-09-29

**Authors:** Ranran Li, Zhihong Zou

**Affiliations:** School of Economics and Management, Beihang University, Beijing 100191, China; E-Mail: liranran1101@163.com

**Keywords:** water environmental capacity, Bayesian approach, inverse method, eutrophication

## Abstract

An integrated approach using the inverse method and Bayesian approach, combined with a lake eutrophication water quality model, was developed for parameter estimation and water environmental capacity (WEC) analysis. The model was used to support load reduction and effective water quality management in the Taihu Lake system in eastern China. Water quality was surveyed yearly from 1987 to 2010. Total nitrogen (TN) and total phosphorus (TP) were selected as water quality model variables. Decay rates of TN and TP were estimated using the proposed approach. WECs of TN and TP in 2011 were determined based on the estimated decay rates. Results showed that the historical loading was beyond the WEC, thus, reduction of nitrogen and phosphorus input is necessary to meet water quality goals. Then WEC and allowable discharge capacity (ADC) in 2015 and 2020 were predicted. The reduction ratios of ADC during these years were also provided. All of these enable decision makers to assess the influence of each loading and visualize potential load reductions under different water quality goals, and then to formulate a reasonable water quality management strategy.

## 1. Introduction

In recent years, the deterioration of surface water bodies is becoming more and more serious, which has become one of the most important environmental issues facing national and local governments worldwide. Therefore, it is very important to make effective water quality management strategies to reduce the resulting environmental pressure and protect the water ecological system. The water environment management system in China has evolved from a concentration control and goal amount control focus to capacity amount control. Water environmental capacity (WEC), as one of the core contents of any capacity amount control system, plays an important role in water quality management. WEC is defined as the maximum load that can be carried by natural waters to meet the required water quality target. There are many kinds of methods to calculate WEC [1,2,3,4,5]. Among them, the formula method is the most basic method, which is deduced under certain conditions according to the WEC definition and the water quality model (WQM). There are also many kinds of WQMs [1,6,7,8]. Therefore, many WEC calculation formulas applying to different conditions can be obtained. The required parameters in the formula are obtained by the WQM. Therefore, WQM is considered very important for water quality management [9,10,11].

In view of the uncertainty of the water environmental system, the unascertained mathematical method is widely used to calculate the WEC [7,12]. In the unascertained mathematical method, water environment parameters in the water system (such as flow rate, pollutant concentration, and pollutant degradation coefficient) are all defined as unascertained numbers and often generated by stochastic simulation. Then, in combination with the WEC model, the unascertained WEC calculation model is established. Possible values of WEC and their credibility are calculated, and then the WEC is obtained. However, errors may be caused during the process of defining parameters as unascertained numbers. Therefore, it will be more reasonable if using the known data to estimate the unknown data. The integration of Bayesian and inverse methods can solve this problem well by getting the posterior distributions of the uncertain parameters according to their prior distributions, and then WEC is obtained under the uncertainty condition.

The prominent advantage of Bayesian statistics is their capability to transform the uncertainty problem into estimated model parameters or loads in terms of a joint posterior distribution. It can easily assess the uncertainty for decision makers. In addition, Bayesian methods incorporate existing expert knowledge and experiences via the prior distribution [13,14], and so can result in more precise estimation than traditional methods do, especially when the observed data are insufficient. Inverse methods have been widely applied to address environmental issues in recent years, including model parameter estimation [10,15,16], groundwater [17,18,19], non-point source estimation [20,21], and wastewater systems [22].

The utility of the integrated approach has been confirmed in many environmental case studies, including ground water quality modeling [23], contaminant source identification [24], shellfish aquaculture ecosystem modeling [25], non-point source load estimation [16], loading estimation and uncertainty assessment [26,27], and the multi-pollution source water quality model [28]. However, this combination has seldom been used in lake eutrophication water quality model and corresponding WEC analysis.

In this study, a Bayesian approach combined with inverse method for the lake eutrophication water quality model was developed to estimate parameters and analyze WEC under uncertainty conditions. Taihu Lake in the Jiangsu Province in eastern China was studied. The model results will help local decision makers reduce and allocate loads in an effective and efficient manner.

## 2. Materials and Methods

### 2.1. Study Area and Data Source

Taihu Lake is located in the south of Jiangsu Province and the south of the Yangtze River Delta. The entire water area of Taihu Lake is in Jiangsu Province. The south lake is adjacent to Zhejiang Province. Taihu Lake is the largest lake in east China. It is also the third largest freshwater lake in China. The area of the lake is 2338 km^2^; the average lake capacity is 44.3 × 10^8^ m^3^; the average surface area is 2425 × 10^6^ m^2^; and the watershed area is 36,895 km^2^. As a storage center of river water resources, Taihu Lake has the functions of flood control, water supply, ecology, shipping, tourism and aquaculture, so its WEC is directly related to the social and economic development of Jiangsu Province, Zhejiang Province and Shanghai City. In 2007, cyanobacteria malignantly bloomed in Taihu Lake, which caused huge economic losses. Given the serious perniciousness of the cyanobacteria blooms, state and local governments have adopted various measures to reduce emissions of pollutants in the basin and restore the lake’s ecological environment. In recent years, the Taihu Lake management has made certain progress. At present, the permanganate index and ammonia nitrogen in Taihu Lake have been obviously improved; total nitrogen (TN) and total phosphorus (TP) are still the main pollutants. A study on Taihu Lake’s environmental capacity has an important significance for Taihu Lake management and the sustainable development of the Taihu Lake basin. Research on Taihu Lake WEC started in the 1990s, and there have been many scholars discussing it over the last twenty years [29,30,31,32,33].

Previous studies were based on deterministic methods. Considering the uncertainty of the water environmental system, the Bayesian and inverse methods are used to get the posterior distributions of the uncertain parameters according to their prior distributions, and then WEC is obtained under uncertainty conditions.

Data from Taihu Lake from 1987 to 2010 was used to estimate the parameters. Monitored parameters included pollutant concentration, and many other important variables. Concentration of TN and TP are derived from routine monitoring data of the environmental protection department [30,32]. Out flow amount and loads of TN and TP are calculated according to routine patrol data of the water conservancy department [31,34]. In this study, a lake eutrophication water quality model and Bayesian statistics were developed for parameters estimation and variable WEC calculation with limited data requirements. The used data refer to Table S1 in the Supplementary Materials.

### 2.2. Lake Eutrophication Water Quality Model

When one takes years as the time scale to study the process of lake eutrophication, lakes often can be seen as a completely mixed reactor. The Vollenweider model [35] is adopted here. This model often does not describe physical, chemical and biological processes in lakes, and also does not consider the thermal stratification of the lake. According to Vollenweider model assumption, the change rate of pollutant concentration in lake is a function of input, output and the deposition amount of this pollutant in the lake, which can be expressed by the following equilibrium equation:

(1) $$\frac{VdC}{dt}=I-QC-SCV$$

where *V* is the storage capacity of the lake (10^8^ m^3^), *C* is the water quality component’s concentration in the lake (t/10^8^ m^3^), *I* is the total load of some nutrient (t/a), *Q* is the out flow amount of the lake (10^8^ m^3^/a), *S* is the decay rate for nutrient in the lake (1/a).

Equation (1) divided by *V* is:

(2) $$\frac{dC}{dt}=\frac{I}{V}-rC-SC$$

where *r* is the hydraulic scour rate (1/a) and $r\text{=}\frac{Q}{V}$.

If *t* = 0, *C* = *C*_0_, then the analytical solution of this equation is:

(3) $$C=\frac{I}{V(r+S)}+\frac{V(r+S)C_{0}-I}{V(r+S)}\exp[-(r+S)t]$$

When *t* → ∞, the balance concentration of nutrient is:

(4) $$C=f(I,V,r,S)=\frac{I}{V(r+S)}$$

The model from Equation (4) can be viewed as a forward model, which is from *S* to *C*. The reverse model for Equation (4) is:

(5) $$S=f^{-1}(V,r)$$

Thus, the problem of decay rate *S* estimation can be transformed into an inverse model with the aim of finding *S* so that defined objective function can be met. The Bayesian approach was then applied for *S* estimation.

According to Equation (4), the water environment capacity can be obtained as follows:

(6) $$WEC=C_{S}V(r+S)$$

where *V*, *r* are as mentioned above, *C_S_* (t/10^8^ m^3^) is the required water quality target, *S* adopts its posterior distribution from the Bayesian model results.

By WEC, the corresponding allowable discharge capacity (ADC) can be got. According to reference [33], the ADC is:

(7) ADC=(WEC−W)/α

where *W* is the uncontrolled amount of pollutants into the lake, including the amount of pollutants into the lake through the river (including water diversion from the Yangtze), atmospheric dry and wet deposition, lake island and seine, α is the coefficient of pollutants into the lake. W_TN_ = 12115 t/a, W_TP_ = 808 t/a, α_TN_ = 0.84, α_TP_ = 0.90, calculated by the historical data in [33].

### 2.3. Bayesian Approach

Bayesian statistics have been increasingly used to address environmental and other scientific issues in recently years [36]. Bayesian methods provide a framework within which all unknown parameters are treated as random variables and their distributions are derived from pre-existing information, then a probability distribution on the parameter space can be obtained, and the uncertainty about the parameters is summarized. Thus, Bayesian statistics provide an important method for uncertainty analysis and present crucial information for management decision making [37]. The Bayes theorem can be written as:

(8) $$P(\text{θ}/X)=\frac{P(X/\text{θ})P(\text{θ})}{P(X)}$$

where *P*(θ / *X*) is the posterior distribution of θ and represents the conditional distribution of the parameter θ values given observed data *X*. In this study, the parameter θ refers to *S*, *i.e.*, θ = {*S*_1_,*S*_2_,…*S*_n_}. *P*(*X*) is the expected value of the likelihood function over the parameter distribution as a normalizing constant. *P*(θ) is the prior distribution of θ based on the observed data. *P*(θ / *X*) is the likelihood function, which describes the mechanistic and statistical relationship between the predictor and variables. While the Bayesian framework provides an attractive approach to parameter estimation and uncertainty analysis, it is impossible obtain the analytically posterior distributions, which hinder the practical application of Bayesian approach. To this end, the Markov chain Monte Carlo (MCMC) algorithm has been applied to obtain the numerical summarization of parameters [36].

There are three major steps in the Bayesian model using the MCMC sampling method [38]: (i) formulating the prior probability distributions for targeted parameters; (ii) specifying the likelihood function; and (iii) MCMC sampling for the posterior probability distributions. Gibbs sampling and Metropolis-Hastings algorithms are popularly used for MCMC sampling. The aim of MCMC sampling is to generate samples from the posterior distribution of the model parameters by simulating a random process, and the process has the posterior distribution as its stationary distribution [39]. Then the random sample can be provided and used for further estimation. In this study, Gibbs sampling was used to address the practical implementation of MCMC via a specialized and free software package, WinBUGS [40].

To specify the likelihood function of *S*, Equation (4) was incorporated into Equation (8). The observed concentration of C* is taken as the modeled value with a normally distributed random error ε, which is formulated by:

(9) $$C^{*}=C+\text{ε}=\frac{I}{V(r+S)}+\text{ε}$$

where ε has zero mean and variance of σ^2^. The value of σ was assumed to follow a Gamma distribution [21] and can be estimated in the Bayesian model based on the prior distribution. Then the likelihood function for all twelve years of observations of Taihu Lake can be expressed as:

(10) $$P(X/\text{θ})=\prod_{i=1}^{24}\frac{1}{\sqrt{2\text{π}{\text{σ}}^{2}}}e^{-\frac{{\left(C^{*}-C\right)}^{2}}{2{\text{σ}}^{2}}}$$

Posterior simulation, based on Bayesian model results for Taihu Lake, was performed to understand the uncertainty associated with WEC.

The MCMC simulations for the TN and TP models were carried out separately in WinBUGS. All years were modeled in the same WinBUGS program. The prior distribution of *S* was determined using interval values from the literature at the range from 1 to 2.5 a^−1^ for TN and the range from 3 to 6 a^−1^ for TP [33]. The MCMC was carried out in WinBUGS with one Markov chain and 50,000 iterations [38]. The WinBUGS code used in this study can be found in the Supplementary Materials.

## 3. Results and Discussion

### 3.1. Estimation of S and Assessment of Model Fit

Posterior distributions of *S_TN_*, *S_TP_* are given in Table 1, including mean, 5%, 25%, 75% and 95% credible level values, the standard deviation and the Monte Carlo (MC) errors (an estimate of the difference between the mean of the sampled values and the true posterior mean). The MC errors for posterior *S_TN_*, *S_TP_* were less than 8% of the SD, indicating that the model converged well [40].

**Table 1:** The posterior decay rate for TN (*S_TN_*, 1/a) and TP (*S_TP_*, 1/a) and their standard deviations (SD) and Monte Carlo (MC) errors of the Taihu Lake.

	5%	25%	Mean	75%	95%	SD	MC Error
*S_TN_*	1.728	1.805	1.861	1.914	2.0	0.08329	0.0003863
*S_TP_*	4.148	4.464	4.698	4.918	5.32	0.3579	0.001441

The posterior distributions shown in the above table are close to the average decay rates calculated from Vollenweider model in [33] and the decay rates used in other studies [41,42,43], so the estimated results are reliable. However, in this study, more historical data were used for parameter seeking than that in [33]. And the decay rate was estimated by the Bayesian method rather than calculating by the average of each year’s data in [33]. Therefore, for complex and uncertain water environment systems, the method in this study is more reasonable.

**Figure 1:**
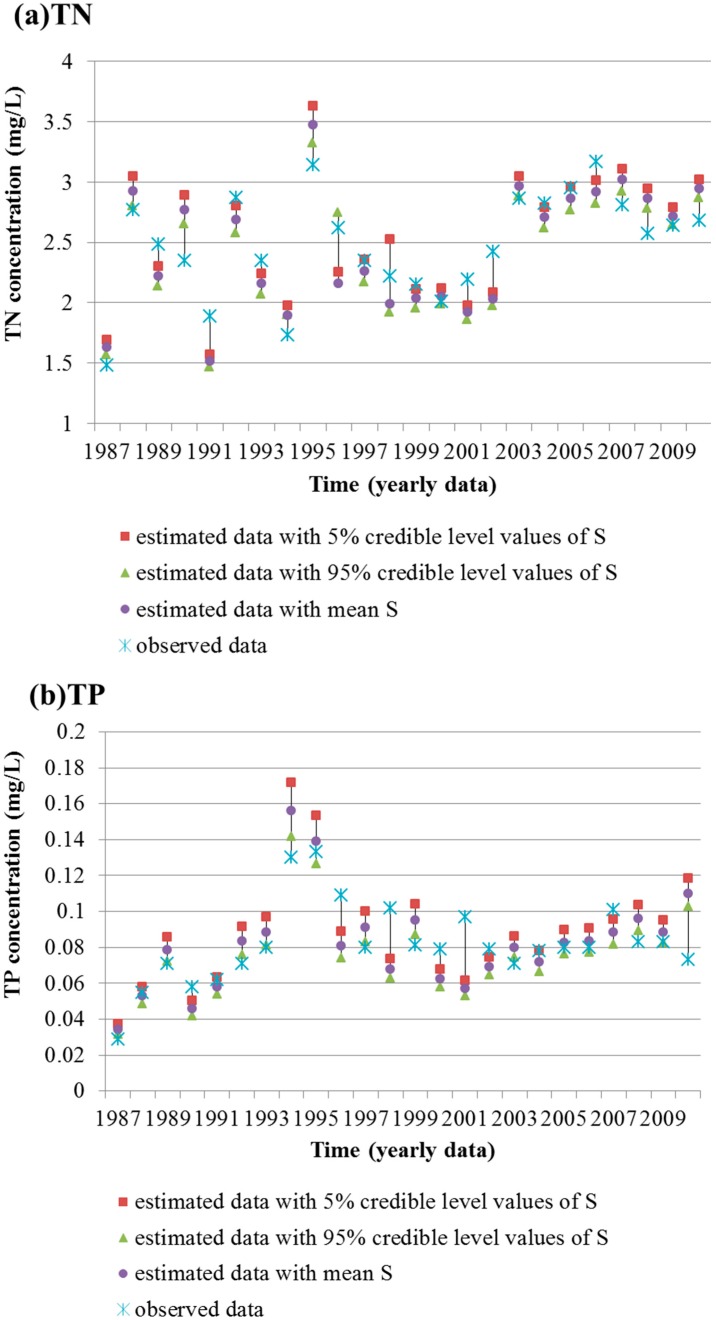
Model fitting results for TN (**a**) and TP (**b**) between observed data and simulated data with mean *S*, 5% and 95% credible level values of *S*.

The observed TN and TP concentrations of the 24 years (1987–2010) were compared with corresponding simulated data. The contrast results are shown in Figure 1. The simulated concentrations and observed ones are strongly correlated with R^2^ > 0.69 (*p* < 0.01) and Nashe-Sutcliffe efficiency [44] > 0.47 for TN, and R^2^ > 0.57 (*p* < 0.01) and Nashe-Sutcliffe efficiency > 0.34 for TP. Thus, the calculation of water quality capacity and according water quality management strategies could be carried out based on the model.

### 3.2. Variable WEC and Required Load Reduction Ratio

The water quality targets (annually average value) for TN and TP of Taihu Lake in 2015 and 2020 are described in Table 2. Under the water quality targets of 2015, WECs in 2011 in Taihu Lake are calculated, using the estimated decay rates in Table 1 and according to the Equation (6). The calculation results are shown in Table 3. The historical loads of TN and TP in 2011 are 58336 t/a and 3308 t/a, which have beyond the WECs at any confidence level. Then load reduction ratios were calculated at different confidence levels. These are also shown in Table 3.

**Table 2:** Water quality Targets (annually average value (mg/L)) for TN and TP.

	2015	2020
TN	2.2 (inferiorV)	2.0 (IV)
TP	0.06 (IV)	0.05 (III)

Note: The figures in parentheses show that the corresponding water quality rank in GB3838-2002.

**Table 3:** WEC and load reduction ratio of Taihu Lake in 2011.

	TN		TP	
	WEC (t/a)	Load Reduction Ratio	WEC (t/a)	Load Reduction Ratio
5%	46743	19.87%	1918	42.02%
25%	47494	18.59%	2002	39.48%
Mean	48040	17.65%	2064	37.60%
75%	48556	16.76%	2123	35.83%
95%	49394	15.33%	2230	32.60%

It is worth noting that the historical loads are all above the WEC Thus, pollutant reduction for TN and TP is necessary to meet the water quality goals. According to Table 3, load reduction ratio of TN is above 15% and load reduction ratio of TP is above 30%. Therefore, both TN and TP loads are quite high and among them the TP pollution is more serious. Therefore, the effectiveness for TN and TP pollution control and management is not obvious. Therefore, the urgency to control the nutrients should be recognized. New nitrogen and phosphorus pollution sources must be prohibited. Management and examination of TN and TP should be strengthened according to the relative plans [45], so as to reach the standard.

**Figure 2:**
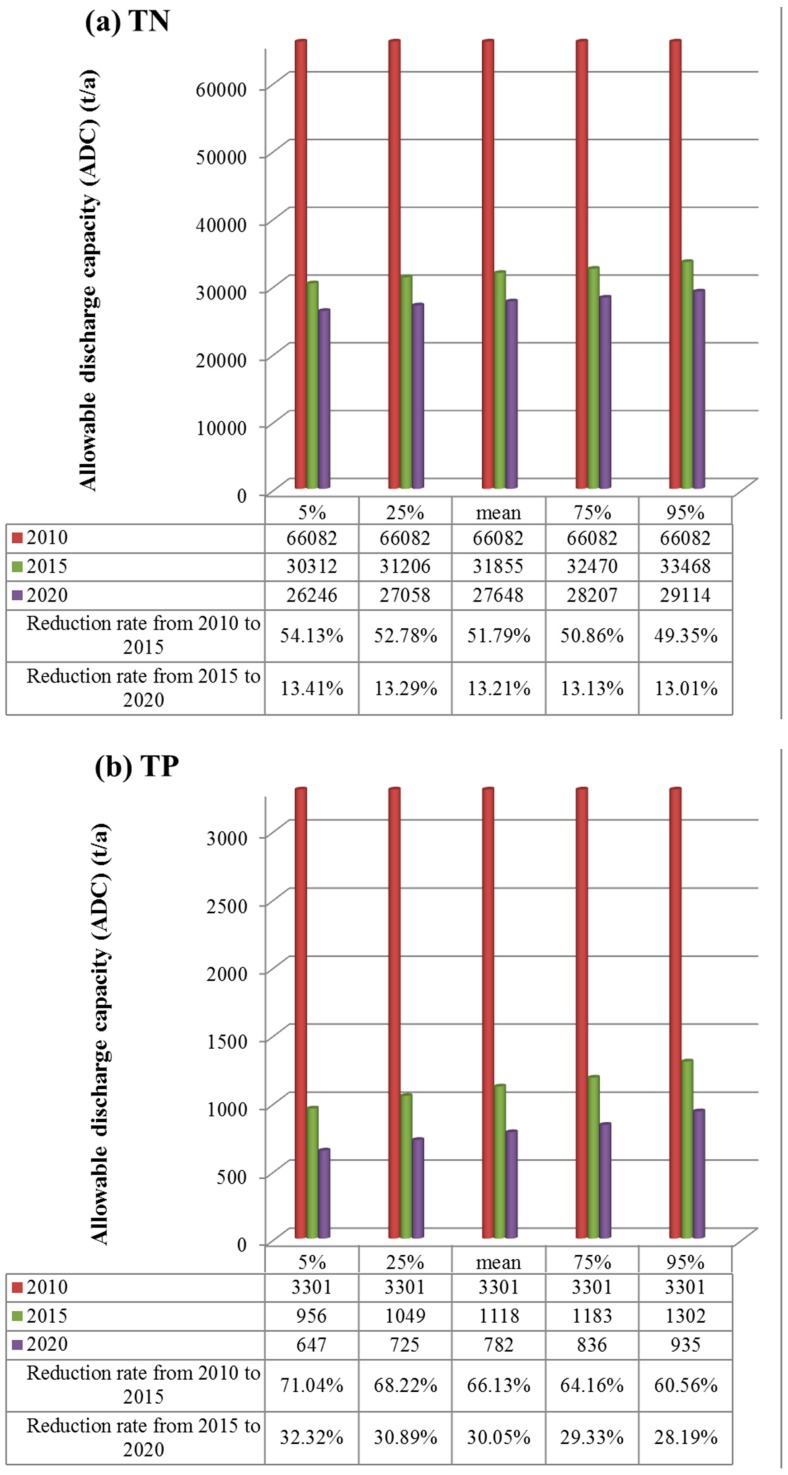
ADC in 2015 and 2020 and corresponding reduction ratio at different confidence levels for TN (**a**) and TP (**b**).

In order to control TN and TP pollution and reduce the emissions reasonably, the WEC in 2015 and 2020 were predicted according to Equation (6), where the average hydraulic scour rate *r* was used, that is, out flow *Q* used the annual average data of 1987–2010. Decay rate *S* used the estimated data. *C_S_* (t/10^8^ m^3^) used the required water quality target in Table 2. The predicted results are shown in Table S2 in the Supplementary Materials. Then, the ADC in 2015 and 2020 can be predicted according to Equation (7) and data in Table S2. As the lake pollutant emissions haven’t changed much in the past few years according to [33], here it is assumed unchangeable in the next few years. The TN and TP emissions of Taihu in 2010 are 66082 t/a and 3301 t/a, respectively. Therefore, the reduction ratio from 2010 to 2015 and from 2015 to 2020 can be calculated. All these are shown in Figure 2. In the figure, the 5%, 25% mean, 75%, and 95% are corresponding to the different confidence levels in Table S2. The ADCs in 2015 and 2020 are predicted by the estimated S. The ADC in 2010 is assured, so it is the same value under different confidence levels.

From Figure 2, it can be seen that from 2010 to 2015 the TN emission reduction ratio is 49%–54%, and the TP emission reduction ratio is 60%–71%, which are both very high. From 2015 to 2020, the reduction ratios of TN and TP are above 13% and 28%–32%, respectively, which both decline when compared with the former five years, but are still not low.

In a word, to achieve the water quality goals is a very difficult task. In addition, over a fairly long period of time, TP and TN concentrations in Taihu Lake will be in an interval which is quite prone to cyanobacteria blooms [46]. It is difficult to fundamentally eliminate the possibility of cyanobacteria blooms. In order to ensure Taihu Lake water quality security, some effective emergency measures, such as “water diversion from the Yangtze” [47], cyanobacteria salvage, should be carried out over a longer period of time.

## 4. Conclusions

An integrated approach of inverse method and Bayesian inference, combined with the lake eutrophication water quality model, was applied to estimate the decay rate of TN and TP of Taihu Lake, China. The model was fitted to water quality monitoring data from 1987 to 2010. The estimated parameters were used to calculate WEC of Taihu Lake in 2011, and the reduction ratios of pollutant loads were given. From the calculated results, the effectiveness for TN and TP pollution control and management is not obvious, and there is still a lot of work to do to meet the water quality target. Therefore, some effective emergency measures should be carried out. The estimated parameters were also used to predict the WEC and ADC in 2015 and 2020, and the reduction ratio of ADC from 2010 to 2015 and the one from 2015 to 2020 were given, respectively. This calculation will provide certain theoretical basis for strategy formulation in the next few years.

The model result is based on annual average data. The model can be improved by incorporating a longer-period dataset for better interpretation of water quality changes and monthly data for seasonal variations. WEC could then be estimated on a seasonal scale or an even shorter period. Then, more detailed water quality management strategies can be made.

## Supplementary Files

Supplementary File 1

## References

[1] Wang H.D., Xia Q. (1983). Advances in environmental capacity. Environ. Sci. Technol..

[2] Yu S. (1984). Study on the environmental capacity. Mar. Environ. Sci..

[3] Xia Q., Sun Y., He Z., Li L.Y., Su Y.B., Deng C.L. (1989). Calculation method summary of total amount control of water pollutant. Res. Environ. Sci..

[4] Xu Z.X., Lu S.Q., Lin W.Q. (2003). Calculating analysis on water environmental capacity of ridal river networks. Shanghai Environ. Sci..

[5] Li R.Z., Wang J.Q., Wang C., Qian J.Z. (2003). Calculation of river environmental capacity under unascertained information. Adv. Water Sci..

[6] Han L.X., Zhu D.S., Yao Q. (2001). Water environment capacity calculating method for shallow-broad rivers. J. Hohai Univ. Nat. Sci..

[7] Li S.W., Li H.Y., Xia J.X. (2005). Dapeng Bay water environment capacity analysis on the base of Delft 3D Model. Res. Environ. Sci..

[8] Dong F., Peng W.Q., Liu X.B., Wu W.Q. (2012). Study on calculation of water environmental capacity of river basin. Water Resour. Hydropower Eng..

[9] Saadatpour M., Afshar A. (2007). Waste load allocation modeling with fuzzy goals simulation-optimization approach. Water Resour. Manag..

[10] Zou R., Lung W.S., Wu J. (2007). An adaptive neural network embedded genetic algorithm approach for inverse water quality modeling. Water Resour. Res..

[11] Qin X.S., Huang G.H., Chen B., Zhang B.Y. (2009). An Interval-Parameter Waste-Load-Allocation Model for river water quality management under uncertainty. Environ. Manag..

[12] Li R.R., Zou Z.H. (2014). Calculation of River Water Environmental Capacity Based on Trapezoidal Fuzzy Number and Stochastic Simulation.

[13] Freni G., Mannina G. (2010). Bayesian approach for uncertainty quantification in water quality modeling: The influence of prior distribution. J. Hydrol..

[14] Neuman S.P., Xue L., Ye M., Lu D. (2012). Bayesian analysis of data-worth considering model and parameter uncertainties. Adv. Water Resour..

[15] Shen J., Kuo A.Y. (1998). Application of inverse method to calibrate estuarine eutrophication model. J. Environ. Eng..

[16] Liu Y., Yang P.J., Hu C., Guo H.C. (2008). Water quality modeling for load reduction under uncertainty: A Bayesian approach. Water Res..

[17] Michalak A.M., Kitanidis P.K. (2004). Estimation of historical groundwater contaminant distribution using the adjoint state method applied to geo-statistical inverse modeling. Water Resour. Res..

[18] Vermeulen P.T.M., Heemink A.W., Valstar J.R. (2005). Inverse modeling of groundwater flow using model reduction. Water Resour. Res..

[19] Snehalatha S., Rastogi A.K., Patil S. (2006). Development of numerical model for inverse modeling of confined aquifer: Application of simulated annealing method. Water Int..

[20] Shen J., Jia J.J., Sisson G.M. (2006). Inverse estimation of nonpoint sources of fecal coliform for establishing allowable load for Wye River, Maryland. Water Res..

[21] Shen J., Zhao Y. (2010). Combined Bayesian statistics and load duration curve method for bacteria nonpoint source loading estimation. Water Res..

[22] Bumgarner J.R., McCray J.E. (2007). Estimating biozone hydraulic conductivity in wastewater soil-infiltration systems using inverse numerical modeling. Water Res..

[23] Woodbury A.D., Ulrych T.J. (2000). A full-Bayesian approach to the groundwater inverse problem for steady state flow. Water Resour. Res..

[24] Michalak A.M., Kitanidis P.K. (2003). A method for enforcing parameter non-negativity in Bayesian inverse problems with an application to contaminant source identification. Water Resour. Res..

[25] Dowd M., Meyer R. (2003). A Bayesian approach to the ecosystem inverse problem. Ecol. Model..

[26] Chen D.J., Lu J., Wang H.L., Shen Y.N., Gong D.Q. (2011). Combined inverse modeling approach and load duration curve method for variable nitrogen total maximum daily load development in an agricultural watershed. Environ. Sci. Pollut. Res..

[27] Chen D.J., Dahlgren R.A., Shen Y.N., Lu J. (2012). A Bayesian approach for calculating variable total maximum daily loads and uncertainty assessment. Sci. Total Environ..

[28] Zhao Y., Sharma A., Sivakumar B., Marshall L., Wang P., Jiang J. (2014). A Bayesian method for multi-pollution source water quality model and seasonal water quality management in river segments. Environ. Modell. Softw..

[29] Su J.Y., Liu S.K., He S.L., Qin P.Y., Zou S.M., Zhai S.H. (1992). Research on Taihu Lake water environmental capacity. J. Hydraul. Eng..

[30] Qian Y.C., He P. (2009). Analysis of water environment variation in the Taihu Lake Basin. Yangtze River.

[31] Yan S.W., Yu H., Zhang L.L., Xu J., Wang Z.P. (2011). Water quantity and pollutant fluxes of inflow and outflow rivers of Lake Taihu, 2009. J. Lake Sci..

[32] Cheng X. (2012). Study on relationship between water quality change and economic development of Taihu Lake. Environ. Sustain. Dev..

[33] Cheng S.T., Qian Y.C., Zhang H.J. (2013). Estimation and application of macroscopic water environmental capacity of total phosphorus and nitrogen for Taihu Lake. Acta Sci. Circumst..

[34] Shen J.Y., Shi Y.D., Gan S.W., Gao Y., Xu F. (2011). Changing trend of water entering western area of Taihu Lake Basin and causal analysis. Water Res. Prot..

[35] Wang B.Z. (1990). Water Pollution Control Engineering.

[36] Qian S.S., Stow C.A., Borsuk M.E. (2003). On Monte Carlo methods for Bayesian inference. Ecol. Model..

[37] Reckhow K.H. (1994). Importance of scientific uncertainty in decision-making. Environ. Manag..

[38] Malve O., Qian S.S. (2006). Estimating nutrients and chlorophyll a relationships in Finnish lakes. Environ. Sci. Technol..

[39] Marshall L., Nott D., Sharma A. (2004). A comparative study of Markov chain Monte Carlo methods for conceptual rainfall-runoff modeling. Water Resour. Res..

[40] Lunn D.J., Thomas A., Best N., Spiegelhalter D. (2000). WinBUGS-a Bayesian modeling framework: Concepts, structure, and extensibility. Stat. Comput..

[41] Pang Y, Lu G.H. (2010). Water Environmental Capacity Calculation Theory and Application.

[42] Huang Y.P. (2001). Taihu Lake Water Environment and Pollution Control.

[43] Qin B.Q., Hu W.P., Chen W.M. (2004). Taihu Lake Water Environment Evolution Process and Mechanism.

[44] Borah D.K., Bera M. (2003). Watershed-scale hydrologic and nonpoint-source pollution models: Review of mathematical bases. Trans. ASAE.

[45] The State Coucil (2008). The Whole Comprehensive Treatment Plan of Taihu Lake Water Environment.

[46] Feng M.W., Wu Y.H., Feng S.X., Wu Y.Y. (2008). Effect of different N/P ratios on algal growth. Ecol. Environ..

[47] Wu H.Y., Zhou D.P., He J., Bao C.K. (2008). Integrated benefit assessment of the project water diversion from Yangtze River to Lake Taihu and discussion on the methodology. J. Lake Sci..

