# Supplementary material for: Water Environmental Capacity Analysis of Taihu Lake and Parameter Estimation Based on the Integration of the Inverse Method and Bayesian Modeling
Source: Int J Environ Res Public Health. 2015 Sep 29;12(10):12212–24. doi: 10.3390/ijerph121012212 (PMC4626964; doi:10.3390/ijerph121012212)
Supplement: Supplementary File 1 [file ijerph-12-12212-s001.pdf]

Water Environmental Capacity Analysis of Taihu Lake and Parameter Estimation Based on the Integration of the Inverse Method and Bayesian Modeling

Table S1. Data of Taihu Lake for parameter estimation and water environment capacity calculation.

Year	Q (10^8 m^3/a)	C_{TN} (t/10^8 m^3)	C_{TP} (t/10^8 m^3)	I_{TN} (t/a)	I_{TP} (t/a)
1987	82.44	148	2.9	26856	993
1988	62.72	277	5.5	42434	1431
1989	80.65	248	7.1	36213	2271
1990	57.35	235	5.8	38726	1213
1991	98.57	189	6.2	27435	1783
1992	59.14	287	7.1	38055	2228
1993	71.68	235	8.0	33214	2476
1994	53.76	173	13	25765	4084
1995	55.56	314	13.3	47948	3666
1996	64.52	262	10.9	31764	2204
1997	66.31	235	8.0	33641	2497
1998	100.36	222	10.2	36324	2095
1999	75.27	215	8.1	32074	2692
2000	105.01	201	7.9	38496	1960
2001	112.99	219	9.7	37496	1830
2002	137.49	242	7.9	44596	2390
2003	129.75	286	7.1	62896	2700
2004	106.86	282	7.8	51196	2265
2005	104.41	295	8.0	53479	2581
2006	100.15	317	8.0	53199	2577
2007	116.76	281	10.1	60096	2868
2008	127.94	257	8.3	60253	3234
2009	144.02	264	8.3	61518	3121
2010	148.43	268	7.3	67972	3925

Table S2. WEC of TN and TP in 2015 and 2020.

	2015		2020	
	WEC_{TN} (t/a)	WEC_{TP} (t/a)	WEC_{TN} (t/a)	WEC_{TP} (t/a)
5%	37577	1668	34161	1390
25%	38328	1752	34843	1460
Mean	38874	1814	35340	1512
75%	39390	1873	35809	1561
95%	40228	1980	36571	1650

Supporting Material I: The WinBUGS code for the TN and TP model of Taihu Lake

model for Taihu Lake TN

```
model
{
K~dnorm(0.0,1.0E-6)I(1,2.5)
for(i in 1:N){
C[i]~dnorm(mu[i],tau)
mu[i]<-I[i]/(K*V+Q[i])
}
tau~dgamma(0.001,0.001)
sigma<-1/sqrt(tau)
}
```

model for Taihu Lake TP

```
model
{
K~dnorm(0.0,1.0E-6)I(3,6)
for(i in 1:N){
C[i]~dnorm(mu[i],tau)
mu[i]<-I[i]/(K*V+Q[i])
}
tau~dgamma(0.001,0.001)
sigma<-1/sqrt(tau)
}
```

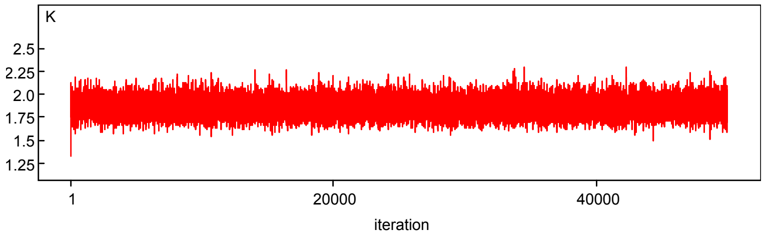

(**a**)

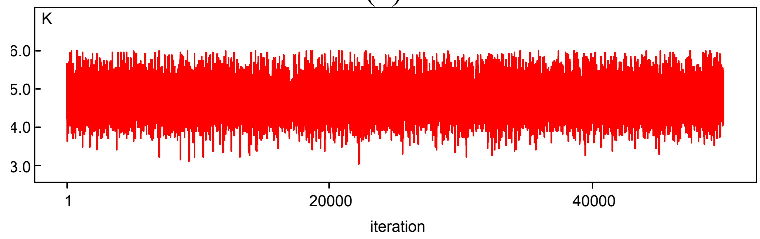

(**b**)

Figure S1. Trace plots of decay rate during MCMC simulation. (**a**) TN; (**b**) TP.

© 2015 by the authors; licensee MDPI, Basel, Switzerland. This article is an open access article distributed under the terms and conditions of the Creative Commons Attribution license (http://creativecommons.org/licenses/by/4.0/).
